# Supplementary material for: Anti-Malignant Ascites Effect of Total Diterpenoids from Euphorbiae ebracteolatae Radix Is Attributable to Alterations of Aquaporins via Inhibiting PKC Activity in the Kidney
Source: Molecules. 2021 Feb 10;26(4):942. doi: 10.3390/molecules26040942 (PMC7916655; doi:10.3390/molecules26040942)
Supplement: Supplementary file 1 [file molecules-26-00942-s001.pdf]

Anti-Malignant Ascites Effect of Total Diterpenoids from *Euphorbiae Ebracteolatae* Radix Is Attributable to Alterations of Aquaporins via Inhibiting PKC Activity in the Kidney

Yuanbin Zhang[1], Dongfang Liu[1], Fan Xue[1], Hongli Yu[1,2,3,4,*], Hao Wu[1,2,3,4,*] Xiaobing Cui[1], Xingde Zhang[1], Hepeng Wang[1]

[1] School of Pharmacy, Nanjing University of Chinese Medicine, Nanjing 210023, China;

[2] Jiangsu Key Laboratory of Chinese Medicine Processing, Nanjing University of Chinese Medicine, Nanjing 210023, China

[3] Engineering Center of State Ministry of Education for Standardization of Chinese Medicine Processing, Nanjing 210023, China

[4] State Key Laboratory Cultivation Base for TCM Quality and Efficacy, Nanjing University of Chinese Medicine, Nanjing 210023, China

[*] Correspondence: Hongli Yu: yuhongli76@163.com Tel.:+025-8679-8281; Hao Wu: whao5795@163.com Tel.: +86-025-8581-1206;

Supplementary Materials

Compound **1** was obtained as a colorless crystal (methanol). The molecular formula $C_{20}H_{26}O_4$ was determined by the positive HR-ESI-TOF-MS. ^{1}H NMR (600 MHz, $CDCl_3$) δ: 4.99 (Sext, 1H), 4.83 (S, 1H), 2.49 (dd, J=18.81, 5.90 Hz, 1H), 2.35 (ddd, J=18.88, 11.60, 7.30 Hz, 1H), 2.19 (d, J=1.88 Hz, 3H), 1.90 (m,1H), 1.65 (m, 3H), 1.47 (br.d, J=13.47 Hz, 1H), 1.40 (m, 1H), 1.24 (br.dd, J=12.84, 1.80 Hz, 1H), 1.19 (td, J=13.47, 3.30 Hz, 1H), 1.14 (s, 3H), 0.95 (s, 3H), 0.89 (s, 3H); ^{13}C NMR (600 MHz, $CDCl_3$) δ: 185.28, 174.05, 161.19, 146.33, 136.55, 133.54, 80.87, 63.79, 50.41, 41.15, 39.75, 34.96, 33.52, 33.03, 24.64, 21.65, 20.28, 18.52, 17.59, 9.87. The ^{1}H and ^{13}C NMR data were in agreement with the reported data [1], named euphorin G.

Compound **2** was obtained as a white powder (methanol). The molecular formula $C_{20}H_{28}O_3$ was determined by the positive HR-ESI-TOF-MS. ^{1}H NMR (600 MHz, $CDCl_3$) δ: 6.33 (s, 1H), 4.92 (s, 1H), 4.51 (s, 1H), 2.54 (d, J=13.44 Hz, 1H), 2.29 (s, 1H), 2.26 (dd, J=13.59, 4.78 Hz, 1H), 1.96 (d, J=12.27 Hz, 1H), 1.88 (s, 3H), 1.85 (d, J=2.16 Hz, 1H), 1.61 (dd, J=7.10, 3.13 Hz, 2H), 1.48 (d, J=13.12 Hz, 1H), 1.39 (qd, J=3.95 Hz, 2H), 1.27 (m, 2H), 0.94 (s, 6H), 0.88 (s, 3H); ^{13}C NMR (600 MHz, $CDCl_3$) δ: 175.44, 152.67, 150.11, 118.15, 113.49, 79.43, 64.53, 60.81, 55.38, 41.73, 40.30, 39.43, 37.10, 33.91, 33.60, 23.78, 21.80, 19.01, 17.34, 8.50. The ^{1}H and ^{13}C NMR data were in agreement with the reported data [2], named ent-11α-hydroxyabicta-8(14),13(15)-dien-16,12-olide。

Compound **3** was obtained as a colorless needle-like crystals ($CHCl_3$). The molecular formula $C_{20}H_{26}O_4$ was determined by the positive HR-ESI-TOF-MS. ^{1}H NMR (600 MHz, $CDCl_3$) δ 4.05 (s, 1H), 3.70 (s, 1H), 2.30 (s, 1H), 2.10 (s, 3H), 2.03 (m, 1H), 1.95 (m, 1H), 1.84 (m, 1H), 1.57 (m, 2H), 1.49 (m, 3H), 1.29 (m, 2H), 1.12 (dd, J = 12.17, 2.32 Hz, 1H), 0.96 (s, 3H), 0.87 (s, 3H), 0.84 (s, 3H); 13C NMR (600 MHz, $CDCl_3$) δ: 169.59, 148.60, 130.26, 85.19, 66.05, 60.94, 55.34, 53.48, 48.01, 41.28, 39.21, 39.15, 35.62, 33.52, 33.47, 21.87, 20.88, 18.42, 15.42, 8.72. The ^{1}H and ^{13}C NMR data were in agreement with the reported data [3], named jolkinolide B。

Compound **4** was obtained as a yellow oil. The molecular formula $C_{19}H_{26}O_2$ was

determined by the positive HR-ESI—TOF-MS. ^{1}H NMR (600 MHz, $CDCl_3$) δ: 6.74 (s, 1H), 5.90 (dd, J=10.7, 17.3, 1H), 4.97 (dd, J=1.14, 17.38 Hz, 1H), 4.90 (dd, J=1.11, 10.79 Hz, 1H), 2.67 (m, 2H), 2.14 (s, 3H), 1.98 (m, 1H), 1.69 (m, 6H), 1.44 (m, 2H), 1.22 (m, 1H), 1.05 (s, 3H), 1.03 (s, 3H) ; ^{13}C NMR (600 MHz, $CDCl_3$) δ: 151.13, 140.78, 140.42, 139.87, 127.11, 122.64, 108.87, 108.79, 39.65, 36.47, 36.45, 36.40, 34.10, 32.90, 26.92, 25.67, 22.80, 21.37, 11.43. The ^{1}H and ^{13}C NMR data were in agreement with the reported data [4], named euphebracteolatin A。

Compound **5** was obtained as a colorless needle-like crystals (methanol). The molecular formula $C_{19}H_{28}O_2$ was determined by the positive HR-ESI-TOF-MS; ^{1}H NMR (600 MHz, $CDCl_3$) δ: 5.89 (s, 1H), 5.78 (dd, J = 17.55, 10.73 Hz, 1H), 4.93 (t, J =7.92 Hz, 2H), 4.88 (d, J = 10.96 Hz, 1H), 1.64 (m, 5H), 1.50 (m, 2H), 1.45 (t, J =13.30 Hz, 1H), 1.26 (ddd, J =13.1, 5.46, 2.51 Hz, 1H), 1.21 (s, 3H), 1.20 (s, 3H), 1.12 (m, 2H), 1.02 (s, 3H), 0.69 (s, 3H); 13C NMR (600 MHz, $CDCl_3$) δ: 183.91, 173.35, 149.97, 117.25, 109.48, 89.90, 43.20, 40.95, 39.58, 39.28, 39.28, 39.09, 35.96, 31.60, 29.99, 28.46, 22.58, 18.86, 18.74. The ^{1}H and ^{13}C NMR data were in agreement with the reported data [5], named fischeria A。

Compound **6** was obtained as a colorless needle-like crystals (methanol). The molecular formula $C_{20}H_{28}O_2$ was determined by the positive HR-ESI-TOF-MS. ^{1}H NMR (600 MHz, $CDCl_3$) δ: 6.28 (s, 1H), 4.83 (dd, J=13.32, 6.00 Hz, 1H), 2.59 (dd, J=13.50, 6.19 Hz, 1H), 2.52 (ddd, J=13.39, 4.10, 2.48 Hz, 1H), 2.20 (m, 2H), 1.90 (d, J=12.63 Hz, 1H), 1.84 (m, 4H), 0.94 (s, 3H), 0.93 (s, 3H), 0.87 (s, 3H); 13C NMR (600 MHz, $CDCl_3$) δ: 175.46, 156.35, 152.36, 116.18, 113.91, 76.08, 55.28, 51.90, 41.93, 41.63, 39.65, 37.18, 33.87, 33.57, 27.53, 23.89, 21.78, 19.08, 16.82, 8.26. The ^{1}H and ^{13}C NMR data were in agreement with the reported data [6], named jolkinolide E。

References

1. Kuang, X.; Li, W.; Kanno, Y.; Yamashita, N.; Kikkawa, S.; Azumaya, I.; Nemoto, K.; Asada, Y.; Koike, K., Euphorins A-H: bioactive diterpenoids from *Euphorbia fischeriana*. *J. Nat. Med.* **2016**, 70, 412-422.
2. Lal, A. R.; Cambie, R. C.; Rutledge, P. S.; Woodgate, P. D., Ent-pimarane and ent-abietane diterpenes from *Euphorbia fidjiana*. *Phytochemistry* **1990**, 29, 2239-2246.
3. Feng, G. Z.; Long, L. Z.; Fang, W. C.; Zhi, L. Q.; Min, S. S.; Mu, L. Z.; Shan, D. S.; Wei, D. Z., Feeding

Deterrents against Two Grain Storage Insects from *Euphorbia fischeriana*. *Molecules* **2011**, 16, 466-476.
4. Mu, S.-Z.; Jiang, C.-R.; Huang, T.; Hao, X.-J., Two New Rosane-Type Diterpenoids from *Euphorbia ebracteolata* Hayata. *Helv. Chim. Acta* **2013**, 96, 2299-2303.
5. Pei, Y.; Koike, K.; Han, B.; Jia, Z.; Nikaido, T., Fischeria A, a novel norditerpene lactone from *Euphorbia fischeriana*. *Tetrahedron Lett.* **1999**, 40, 951-952.
6. Crespi-Perellino, N.; Garofano, L.; Arlandini, E.; Pinciroli, V., Identification of New Diterpenoids from *Euphorbia calyptrata* Cell Cultures. *J. Nat. Prod.* **1996,** 59, 773-776.
